# The role of postprandial very-low-density lipoprotein in the development of atrial remodeling in metabolic syndrome

**DOI:** 10.1186/s12944-020-01386-5

**Published:** 2020-09-22

**Authors:** Hsiang-Chun Lee, Shyi-Jang Shin, Jih-Kai Huang, Ming-Yen Lin, Yu-Hsun Lin, Liang-Yin Ke, He-Jiun Jiang, Wei-Chung Tsai, Min-Fang Chao, Yi-Hsiung Lin

**Affiliations:** 1 Center for Lipid Biosciences, Kaohsiung Medical University Hospital, Kaohsiung, Taiwan; 2 Lipid Science and Aging Research Center, College of Medicine, Kaohsiung Medical University, Kaohsiung, Taiwan; 3 Division of Cardiology, Department of Internal Medicine, Kaohsiung Medical University Hospital, Kaohsiung Medical University, Kaohsiung, Taiwan; 4 Department of Internal Medicine, School of Medicine, College of Medicine, Kaohsiung Medical University, Kaohsiung, Taiwan; 5 Institute/Center of Medical Science and Technology, National Sun Yat-sen University, Kaohsiung, Taiwan; 6 School of Medicine, College of Medicine, Kaohsiung Medical University, Kaohsiung, Taiwan; 7 Division of Nephrology, Department of Internal Medicine, Kaohsiung Medical University Hospital, Kaohsiung Medical University, Kaohsiung, Taiwan; 8 Department of Metabolism, Affiliated Hospital of Kaohsiung Medical University, Kaohsiung, Taiwan; 9 College of Medicine, I-Shou University, Kaohsiung, Taiwan; 10 Department of Medical Imaging, Kaohsiung Medical University Hospital, Kaohsiung Medical University, Kaohsiung, Taiwan

**Keywords:** Atrial cardiomyopathy, Atrial fibrillation, Metabolic syndrome, Negatively charged, Postprandial, Very-low-density lipoprotein

## Abstract

**Background:**

Negatively charged very-low-density lipoprotein (VLDL-χ) in metabolic syndrome (MetS) patients exerts cytotoxic effects on endothelial cells and atrial myocytes. Atrial cardiomyopathy, manifested by atrial remodeling with a dilated diameter, contributes to atrial fibrillation pathogenesis and predicts atrial fibrillation development. The correlation of VLDL-χ with atrial remodeling is unknown. This study investigated the association between VLDL-χ and remodeling of left atrium.

**Methods:**

Consecutively, 87 MetS and 80 non-MetS individuals between 23 and 74 years old (50.6% men) without overt cardiovascular diseases were included in the prospective cohort study. Blood samples were collected while fasting and postprandially (at 0.5, 1, 2, and 4 h after a unified meal). VLDL was isolated by ultracentrifugation; the percentile concentration of VLDL-χ (%) was determined by ultra-performance liquid chromatography. The correlations of left atrium diameter (LAD) with variables including VLDL-χ, LDL-C, HDL-C, triglycerides, glucose, and blood pressure, were analyzed by multiple linear regression models. A hierarchical linear model was conducted to test the independencies of each variable’s correlation with LAD.

**Results:**

The mean LAD was 3.4 ± 0.5 cm in non-MetS subjects and 3.9 ± 0.5 cm in MetS patients (*P* < 0.01). None of the fasting lipid profiles were associated with LAD. VLDL-χ, BMI, waist circumference, hip circumference, and blood pressure were positively correlated with LAD (all *P* < 0.05) after adjustment for age and sex. Significant interactions between VLDL-χ and blood pressure, waist circumference, and hip circumference were observed. When adjusted for obesity- and blood pressure-related variables, 2-h postprandial VLDL-χ (mean 1.30 ± 0.61%) showed a positive correlation with LAD in MetS patients. Each 1% VLDL-χ increase was estimated to increase LAD by 0.23 cm.

**Conclusions:**

Postprandial VLDL-χ is associated with atrial remodeling particularly in the MetS group. VLDL-χ is a novel biomarker and may be a therapeutic target for atrial cardiomyopathy in MetS patients.

**Trial registration:**

ISRCTN 69295295. Retrospectively registered 9 June 2020.

## Background

Metabolic syndrome (MetS) is a major factor associated with the morbidity and mortality of cardiovascular diseases, including atrial fibrillation (AF) [1]. The association of MetS with AF has been identified in epidemiological studies [2–5]. Furthermore, some manifestations of MetS, such as hypertension, diabetes mellitus (DM), and obesity, are independently associated with AF and are attributed to pathogenic mechanisms of atrial remodeling and the development of AF [4–8]. Nevertheless, the findings from clinical studies of the correlation between dyslipidemia and AF are not consistent [9].

Elevated triglycerides are common in MetS and correlate with elevated cardiovascular risk [10]. Triglycerides are primarily carried by very low density lipoprotein (VLDL) in circulation [11]. In the postprandial state, the triglyceride level varies greatly and is affected largely by VLDL-related metabolism [12–14]; therefore, hypertriglyceridemia is preferentially defined by fasting triglyceride values [15]. Nevertheless, nonfasting triglyceride values have been shown to better predict cardiovascular disease risks [16, 17]. The capacity to retain hemostasis upon food intake has been proposed to be important in normal human lipid and glucose metabolism [18]; a sequence of responses is elicited from the gastrointestinal system involving hormones and digestion enzymes, followed by nutritional absorption and delivery to organs and tissues. Abnormalities in postprandial lipid catabolism have been shown to be able to effectively detect early MetS [19].

Furthermore, VLDL remnants in the postprandial state have higher affinity for VLDL receptors [20]. VLDL receptors are expressed abundantly in the heart and are associated with hypertension-related ventricular hypertrophy [21]. Regarding atrial remodeling, we are the first group to examine VLDL-induced atrial remodeling and related mechanisms [9, 22–25]. Of note, the aforementioned changes can be induced only by VLDL in MetS patients but not VLDL in non-MetS subjects. On the basis of electrical charge (i.e., the abundance of negative charge), our colleagues developed a method for stratification of lipoproteins and found much higher cytotoxicity associated with VLDL in MetS patients with the highest abundance of negative charge [23, 26]. Here, we tested the hypothesis that VLDL may be attributed to atrial remodeling, more so in the postprandial state than in the fasting state. We also hypothesized that negatively charged VLDL (referred to as VLDL-χ) may be associated with atrial remodeling in MetS patients.

Left atrium (LA) diameter, a simple echocardiographic parameter, predicts AF recurrence risk after electrical cardioversion and is the first reported index of structural remodeling and a widely accepted predictive marker for AF [27]. On the other hand, noninvasive electrocardiography P wave indexes, such as the P wave duration, PR interval, and terminal force of P waves, have been reported as indicators of atrial electrical dysfunction [28–30]. The objectives of the current study were to determine the quantitative changes in lipids and glucose levels before and after meal intake and to evaluate the associations of VLDL-χ and markers of MetS with indexes of LA remodeling. The differences in postprandial variation in VLDL-χ between patients with and without LA remodeling were also evaluated.

## Methods

### Study participant enrollment

To determine the differences between MetS and non-MetS subjects, regarding of changes in lipids and glucose levels before and after meal intake, the associations by MetS of LA remodeling, and the correlation of VLDL-χ with LA remodeling, this prospective cohort study was conducted at a single Medical Center. Consecutively, 167 participants were enrolled (age range, 23–74 years old). The exclusion criteria included significant coronary heart disease, myocardial infarction, congenital heart diseases, heart failure, significant heart valve diseases, cerebrovascular diseases, cancers, insulin therapy, women with pregnancy or breastfeeding. The inclusion of MetS participants shall meet requirement of criteria with any 3 of the following components: (1) central obesity (waist ≥80 cm for women and ≥ 90 cm for men); (2) raised blood pressure (BP) (systolic BP ≥130 mmHg or diastolic BP ≥85 mmHg or treatment of previously diagnosed HTN); (2) raised fasting glucose (≥100 mg/dL or diagnosed type 2 DM); (3) raised triglycerides (≥150 mg/dL or on triglyceride-lowering treatment); and (4) reduced HDL-C (< 50 mg/dL for women and < 40 mg/dL for men). Among these participants, 87 and 80 had and did not have MetS, respectively. All study participants with MetS were seen by cardiologists or endocrinologists at Kaohsiung Medical University Hospital (Kaohsiung, Taiwan). The study protocol was approved by the Kaohsiung Medical University Hospital Institutional Review Board (IRB) (KMUHIRB-E(I)-20170256). An informed consent form was signed and returned by all participants before joining the study and undergoing plasma collection. This study adhered to the principles of the Declaration of Helsinki.

### Sample and demographic data collection

All study participants were instructed to fast before beginning at midnight and came to the hospital at 8 AM. Each participant underwent two venous blood draws (20 mL for each sampling, in BD VACUETTE® EDTA Blood Tubes (Becton, Dickinson and Company, Franklin Lakes, NJ, USA)); one blood draw was performed in a fasting state, and the other was after finishing the combo meal of 620 cal including a ham-egg sandwich, hashed browns, and a sweetened tea. To abide by the IRB approved protocol, in which the amount of blood drawing shall not exceed 40 mL over a week, a postprandial time was randomly selected for each participant from one of 0.5 h, 1 h, 2 h, and 4 h. In addition to blood sample collection, each participant underwent measurements of height, body weight, abdominal and hip circumferences, blood pressure and heart rate measurements. The medical record, if available, was reviewed, data related to medical history (hypertension and type 2 DM) and medication use were recorded.

### Laboratory testing for biochemical indicators and the quantification of negatively charged low-density lipoprotein (LDL-χ) and negatively charged VLDL (VLDL-χ)

The analysis of biochemical parameters was performed in the Department of Laboratory Medicine at Kaohsiung Medical University Hospital according to the standard operating procedures. Technicians who performed tests were blinded to participants identity and data. Randomly duplicate samples were used to determine the analytical accuracy and measurement precision. As described previously, pairs of plasma samples (in fasting and postprandial states) were obtained from 167 participants. Plasma samples were immediately supplemented with the following after collection to prevent bacterial contamination and oxidation: protease inhibitor cocktail (Roche Diagnostics, Indianapolis, IN), 1% penicillin/streptomycin/neomycin mixture (Invitrogen, Carlsbad, CA), and 0.5 mM EDTA. Plasma LDL and VLDL were isolated by using sequential potassium bromide density-gradient ultracentrifugation between a density range of 1.006 and 1.063 g/mL. Fractions of LDL and VLDL samples that were isolated by density were resolved into subfractions with most negatively charged lipoproteins, i.e., LDL-χ and VLDL-χ, respectively, by increasing the negative charge on UnoQ12 columns (BioRad, Hercules, CA) in the ion-exchange fast-protein liquid chromatography system (FPLC; GE Healthcare, Chicago, IL), as described previously [26, 31]. In short, the columns were first equilibrated with buffer A (0.02 M Tris–HCl, pH 8.0; 0.5 mM EDTA). With a multistep linear gradient of buffer B (1 M NaCl in buffer A) at a flow rate of 2 mL/min under observation at 280 nm, the LDL-χ and VLDL-χ subfractions were eluted, separately concentrated by using Centriprep filters (YM-30; EMD Millipore Corp., Billerica, MA) and sterilized by being passed through 0.22-μm filters. The protein concentration of the LDL and VLDL samples was measured by using the Lowry method [31, 32].

### Electrocardiographic (ECG) parameters

Twelve-lead ECG was performed by experienced medical technicians. The parameters that were measured and recorded by one experienced technician who was blinded to the other data and clinical information included P wave durations, PR intervals, QRS width, QTc intervals, and the terminal force of P waves in lead V1 [33]. Cardiac rhythms were interpreted, and any rhythm other than regular sinus rhythm (such as AF and/or flutter, pacemaker rhythm, ventricular tachycardia, supraventricular tachycardia, and second- or third-degree atrial-ventricular block) was discarded before further analyses.

### Echocardiographic assessment

Echocardiography was performed by one experienced cardiologist using a transthoracic cardiac probe (Vivid 7; General Electric Medical Systems, Horten, Norway), with the participant in the left decubitus position. Two-dimensional and two-dimensional-guided M-mode images were obtained, and LA diameter, left ventricle (LV) size, and LV function were assessed according to the standards of the American Society of Echocardiography [34]. LV ejection fraction (LVEF) was derived by the modified Simpson’s method [34]. The raw data were measured and recorded while the researchers were blinded to the other data.

### Statistical analysis

All continuous variables are presented as the mean ± standard deviation. For all parameters examined in this study, the Shapiro-Wilk normality test was used to determine whether a random sample of values followed a normal distribution. To compare differences between the non-MetS and MetS groups, Student’s t test was used for continuous data, and a chi-square test or Fisher’s exact test was used for binary data. The associations of LA diameter and ECG parameters with body mass index (BMI), waist circumference, hip circumference, systemic blood pressure, pulse rate, fasting and postprandial plasma glucose, triglycerides, HDL-C, LDL-χ and VLDL-χ were evaluated by using the Pearson or Spearman (nonparametric) correlation. To evaluate the determining and confounding factors for LA diameter, simple linear regression and hierarchical multivariable linear regression with the stepwise method in the MetS group, non-MetS group, and total group were used to analyze all the variables, including age, hypertension, DM, BMI, waist circumference, hip circumference, heart rate, systolic blood pressure, diastolic blood pressure, VLDL-χ, triglycerides, high-density lipoprotein cholesterol, LV size, LVEF, E/E’ med, and E/E’ lat. To validate robustness of main findings, several procedures were also carried out. First, a matching procedure by random selection of subjects from the non-MetS group by age within ±2 years was conducted in order to reduce imbalance of age distribution between two groups. Second, multivariable analyses were re-performed by MetS for the overall and the age-matched 47 pairs subjects. Results were considered statistically significant based on a *P* value < 0.05. Statistical analyses were performed by using the statistical package in GraphPad Prism (version 8; GraphPad Software, Inc., San Diego, CA, USA) software system and SPSS statistical software (version 22; IBM Corp., Armonk, NY, USA) and SAS 9.4 software (SAS Institute Inc., Cary, NC, USA).

## Results

### Subject characteristics

Among 80 non-MetS and 87 MetS subjects participating in this study, sex was matched between groups (Table 1). The MetS group was older than the non-MetS group (52.7 ± 11.5 years vs. 44.4 ± 10.2 years, *P* < 0.0001). Among 87 MetS subjects, 83.9 and 78.2% had hypertension and DM, respectively. Markers of obesity, including BMI, waist circumference, and hip circumference, were greater in the MetS group. Similarly, blood pressure and heart rate were higher in the MetS group. In regard to biochemistry data, plasma hemoglobin A1c and fasting glucose were higher in the MetS group. While creatinine was similar in both groups, aspartate aminotransferase (AST) and alanine aminotransferase (ALT) were higher in the MetS group. In regard to lipid profiles, while the MetS group had higher fasting triglycerides and HDL-C, total cholesterol and LDL-C were similar in both groups. In addition, the MetS group had a higher plasma level of uric acid.

**Table 1:** Demographics of the study population

Variables	Non-MetS (*n* = 80)	MetS (*n* = 87)	*P* value
Age, years	44.4 ± 10.2	52.7 ± 11.5	< 0.0001
Male, n (%)	39 (48.1)	46 (52.9)	0.4477
Hypertension, n (%)	0 (0)	73 (83.9)	–
Diabetes mellitus, n (%)	5 (6.2)	68 (78.2)	< 0.0001
BMI, kg/m^2^	22.3 ± 3.0	29.2 ± 4.7	< 0.0001
Waist circumference, cm	75.7 ± 8.2	97.6 ± 9.7	< 0.0001
Hip circumference, cm	94.3 ± 6.9	104.0 ± 9.2	< 0.0001
Systolic BP, mmHg	116.0 ± 9.0	143.5 ± 21.3	< 0.0001
Diastolic BP, mmHg	70.1 ± 7.3	85.9 ± 15.1	< 0.0001
Heart rate, beats/min	75.3 ± 10.0	84.2 ± 12.8	< 0.0001
Biochemistry			
HbA1c, %	5.4 ± 0.2	6.7 ± 0.9	< 0.0001
AST, IU/L	17.7 ± 7.1	24.8 ± 8.9	< 0.0001
ALT, IU/L	20.4 ± 5.4	32.0 ± 18.4	< 0.0001
Creatinine, mg/dL	0.7 ± 0.2	0.8 ± 0.2	0.3018
Glucose, mg/dL	86.1 ± 9.5	119.8 ± 26.4	< 0.0001
Total cholesterol, mg/dL	187.9 ± 35.0	187.6 ± 30.7	0.9598
HDL-C, mg/dL	67.7 ± 14.4	41.5 ± 9.0	< 0.0001
LDL-C, mg/dL	116.5 ± 30.9	124.7 ± 27.6	0.1258
Triglycerides, mg/dL	85.6 ± 27.4	208.7 ± 100.3	< 0.0001
Uric acid, mg/dL	4.9 ± 1.1	5.8 ± 1.3	< 0.0001

*BMI* BOdy mass index, *BP* Blood pressure, *AST* Aspartate aminotransferase, *ALT* Alanine aminotransferase, *HDL-C* High-density lipoprotein cholesterol, *LDL-C* Low-density lipoprotein cholesterol

### Changes in glucose and triglycerides from fasting to after the unified meal in the MetS and non-MetS groups

**Fig. 1:**
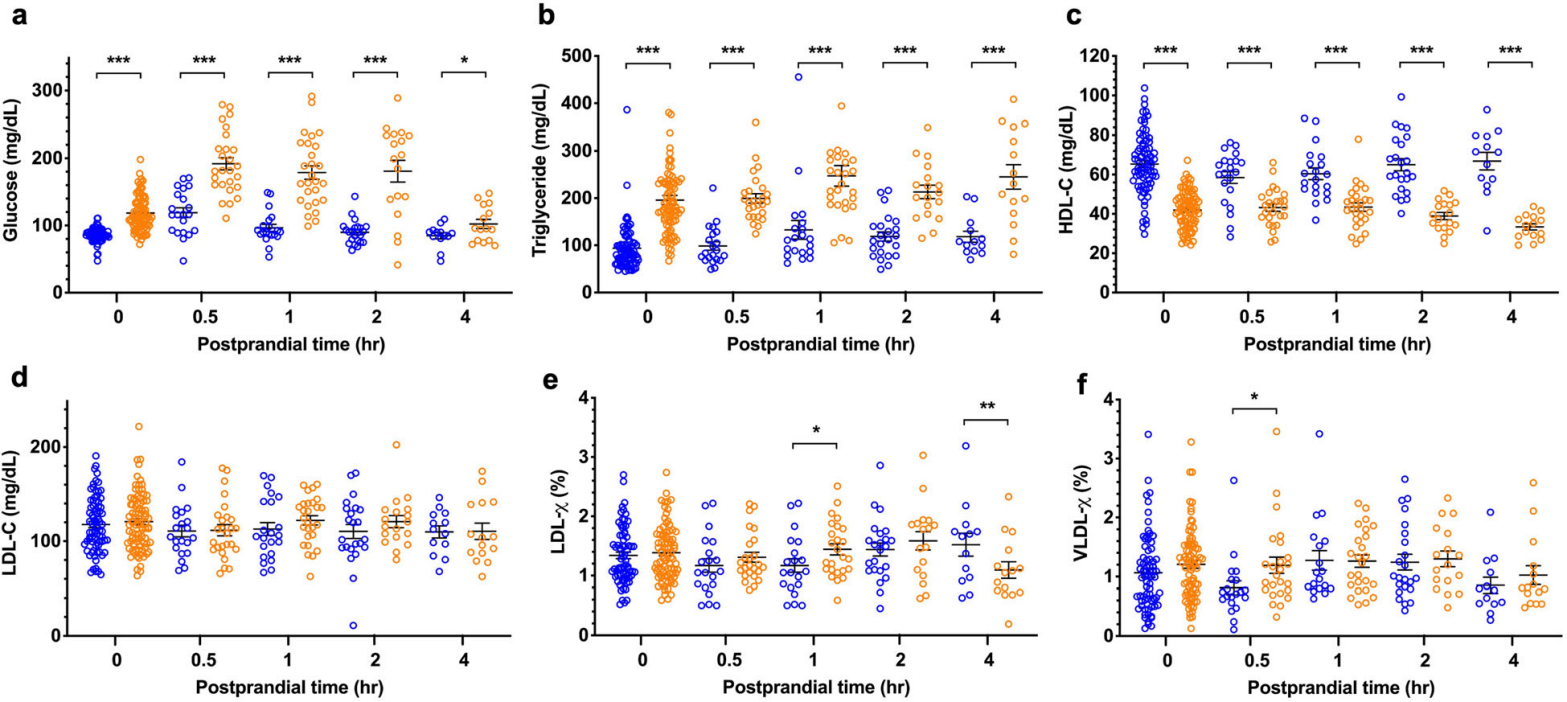
Plasma concentrations of glucose and lipids before (0 h) and after the unified meal (0.5-h, 1-h, 2-h, and 4-h) in the group of healthy controls (in blue) and the group of individuals with metabolic syndrome (in brown). Significant differences between groups are shown as follows: *P* < 0.001 as ***, *P* < 0.01 as **, and *P* < 0.05 as *

Glucose and triglycerides were higher in the MetS group under fasting conditions, while glucose levels decreased 4 h after the meal, and triglyceride levels were still elevated in the MetS group (Fig. 1a and b).

### Changes in HDL-C, LDL-C, LDL-χ and VLDL-χ from fasting to after the unified meal in the MetS and non-MetS groups

HDL-C was lower in the MetS group at fasting and exhibited a decreasing trend after the meal at 2 h and 4 h (Fig. 1c), whereas the postprandial decrease in HDL-C was not present in the non-MetS group. Unlike the results for HDL-C, the plasma LDL-C at fasting and the change after a meal were similar in both groups (Fig. 1d). While LDL-C did not change after a meal, the negatively charged LDL and LDL-χ in the MetS group decreased 4 h after the meal (Fig. 1e). The decrease at 4 h in LDL-χ did not occur in the non-MetS group. At fasting (0 h, Fig. 1f), the negatively charged VLDL-χ was similar in both groups but was significantly higher in the MetS group than in the non-MetS group 0.5 h after the meal. VLDL-χ decreased 4 h after the meal in both groups.

### LA dilation as a marker for atrial structural remodeling

Among 167 non-MetS and MetS subjects participating in this study, subjects of age between 40 and 60 years were recruited to undergo 12-lead ECG and standard echocardiography. The characteristics of 62 non-MetS and 68 MetS subjects who had completed the heart examinations are shown in Table 2. Similar to the overall subjects, the MetS group was more obese, had higher blood pressure, and had a higher heart rate than the non-MetS group. Regarding ECG parameters, the PR interval was wider in the MetS group than in the non-MetS group (167.6 ± 20.0 msec vs. 156.2 ± 24.9 msec, *P* = 0.0064), whereas the P wave duration, terminal force of P waves, QRS width and QTc interval were similar in both groups. Regarding echocardiography, LV function was similar in both groups (LVEF, MetS 67.3 ± 7.1% vs. non-MetS 63.9 ± 11.3%, *P* = 0.0515), whereas the diameter of the aorta, LA and size of LV were larger in the MetS group (Table 2). The ratios of mitral peak velocity of early filling (E) to early diastolic mitral annular velocity (E’) as markers for diastolic function and indicators for LV filling pressure were higher in the MetS group (Table 2). The aforementioned results showed evidence of LA and LV remodeling in MetS participants in this study, and this remodeling was associated with diastolic dysfunction of the LV without overt systolic dysfunction.

**Table 2:** Characteristics of subjects included in the echocardiographic study

Variables	Non-MetS (*n* = 62)	MetS (*n* = 68)	*P* value
Age, years	48.7 ± 7.0	51.9 ± 9.4	0.0351
Male, n (%)	31 (50.0)	37 (54.4)	0.4669
Hypertension, n (%)	0 (0)	58 (85.3)	–
Diabetes mellitus, n (%)	5 (8.1)	49 (72.1)	< 0.0001
BMI, kg/m^2^	22.7 ± 3.0	29.0 ± 3.8	< 0.0001
Waist circumference, cm	78.2 ± 8.5	97.8 ± 9.7	< 0.0001
Hip circumference, cm	95.2 ± 6.5	103.9 ± 8.6	< 0.0001
Systolic BP, mmHg	116.4 ± 10.3	142.8 ± 20.7	< 0.0001
Diastolic BP, mmHg	71.2 ± 7.9	87.2 ± 14.4	< 0.0001
Heart rate, beats/min	73.9 ± 9.2	85.0 ± 13.8	< 0.0001
Electrocardiogram Parameters			
P, msec	105.6 ± 14.2	108.9 ± 13.2	0.1872
PR, msec	156.2 ± 24.9	167.6 ± 20.0	0.0064
Terminal force of P, mVS	1.6 ± 1.5	1.7 ± 1.6	0.8157
QRS, msec	85.7 ± 10.8	88.5 ± 16.5	0.2735
QTc, msec	405.0 ± 21.5	405.5 ± 37.8	0.9292
Echocardiographic Parameters			
Aorta diameter, cm	3.2 ± 0.3	3.4 ± 0.4	0.0012
LA diameter, cm	3.4 ± 0.5	3.9 ± 0.5	< 0.0001
LA volume, mL	48.9 ± 15.4	70.6 ± 18.2	< 0.0001
LVEDD, cm	4.6 ± 0.5	4.8 ± 0.5	0.0049
LVEDV, mL	95.9 ± 30.4	126.0 ± 37.2	< 0.0001
LV mass, gram	107.7 ± 23.7	133.7 ± 28.8	< 0.0001
EF, %	63.9 ± 11.3	67.3 ± 7.1	0.0515
E/E’ (med)	7.7 ± 2.3	9.8 ± 2.7	< 0.0001
E/E’ (lat)	5.8 ± 2.2	6.7 ± 1.8	0.0169
Valve disease, n (%)	0	0	–

*BMI* Body mass index, *BP* Blood pressure, *LA* Left atrium, *LVEDD* Left ventricle end-diastolic dimension, *LVEDV* Left ventricle end-diastolic volume, *LVEF* Left ventricle ejection fraction, *E/E’ (med)* The ratio of mitral peak velocity of early filling (E) to early diastolic mitral annular velocity (E’) at medial mitral ring, *E/E’ (lat)* The ratio of mitral peak velocity of early filling (E) to early diastolic mitral annular velocity (E’) at the lateral mitral ring

### LA dilation was associated with central obesity and hypertension

**Fig. 2:**
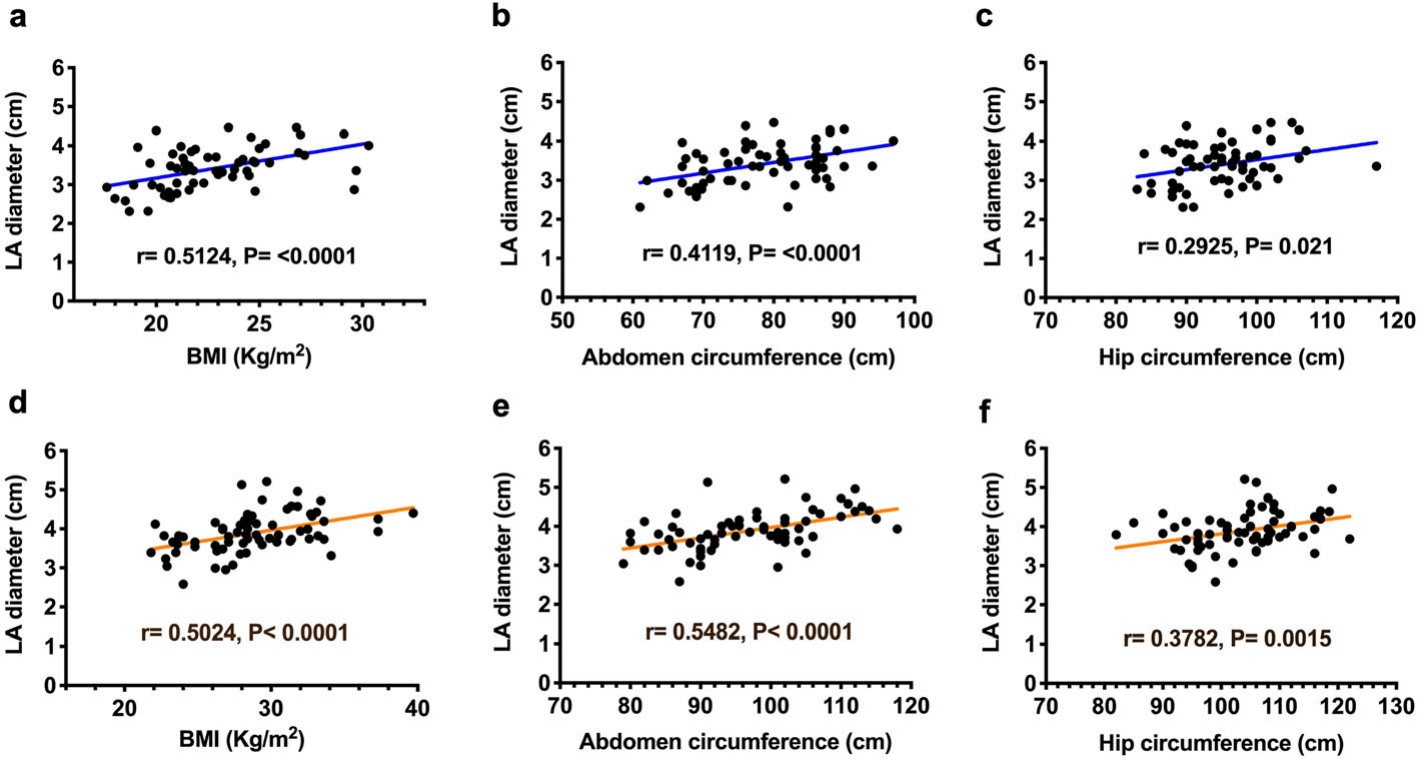
Correlation between left atrial (LA) diameter and the parameters of body size and central obesity body mass index and waist and hip circumferences in individuals without (in blue, **a**-**c**) and with metabolic syndrome (in brown, **d**-**f**). Each correlation factor and *P* value are labeled

**Fig. 3:**
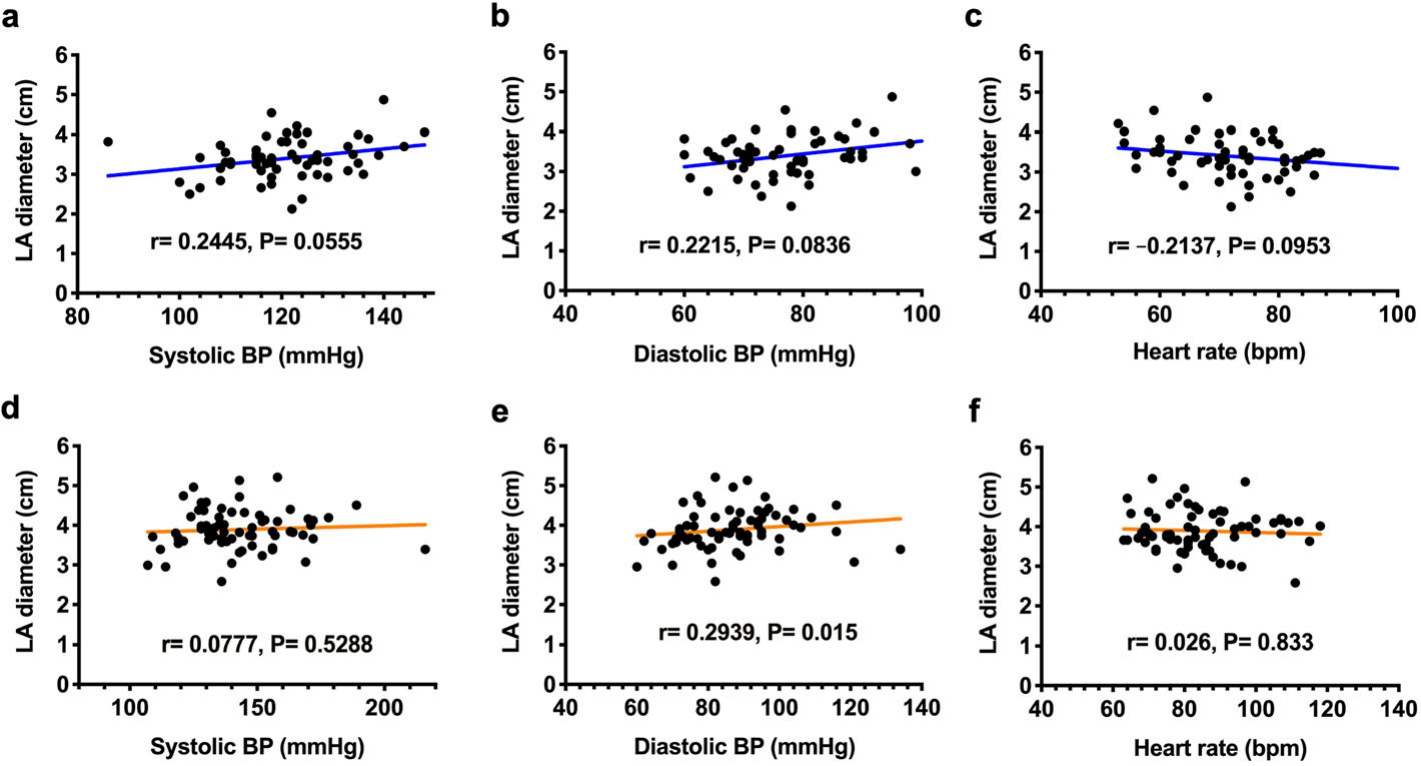
Correlation between left atrial (LA) diameter and systolic and diastolic blood pressure (BP) and heart rate in individuals without (in blue, **a**-**c**) and with metabolic syndrome (in brown, **d**-**f**). Each correlation factor and *P* value are labeled

The LA diameter was shown to significantly correlate with BMI and waist and hip circumferences in both the non-MetS (Fig. 2a-c) and MetS (Fig. 2d-f) groups. The LA diameter was also positively correlated with blood pressure (Fig. 3) and with diastolic blood pressure in the MetS group (Fig. 3e). There was no significant correlation to LA diameter as for either plasma glucose (Suppl. Figure 1), triglyceride (Suppl. Figure 2), VLDL-cholesterol (Suppl. Figure 3), or LDL-χ (Suppl. Figure 4).

### Correlation of LA size with fasting and postprandial VLDL-χ

**Fig. 4:**
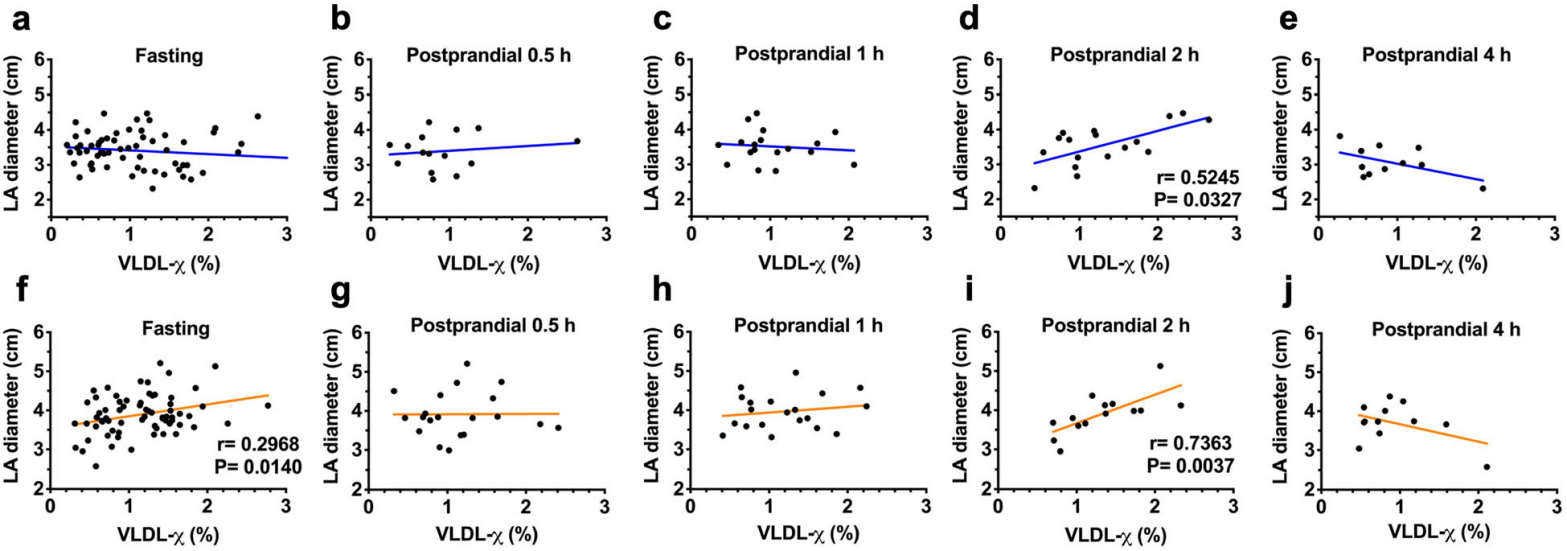
Correlation between left atrial (LA) diameter and negatively charged very-low-density lipoprotein as a percentage (VLDL-χ) before (fasting) and after breakfast (postprandial 0.5-h, 1-h, 2-h, and 4-h) in individuals without (in blue, **a**-**e**) and with metabolic syndrome (in brown, **f**-**j**). A significant correlation (with *P* < 0.05) is indicated with each correlation factor and *P* value

The results of the analysis of the correlation between LA diameter and fasting and 0.5-h, 1-h, 2-h, and 4-h postprandial VLDL-χ are shown in Fig. 4. In the MetS group, the LA size was significantly correlated with fasting (*P* = 0.014; Fig. 4f) and 2-h postprandial VLDL-χ (*P* = 0.004; Fig. 4i). Similarly, the LA size was also significantly correlated with 2-h postprandial VLDL-χ in the non-MetS group (*P* = 0.033; Fig. 4d).

### Two-hour postprandial VLDL-χ is correlated with P wave duration

**Fig. 5:**
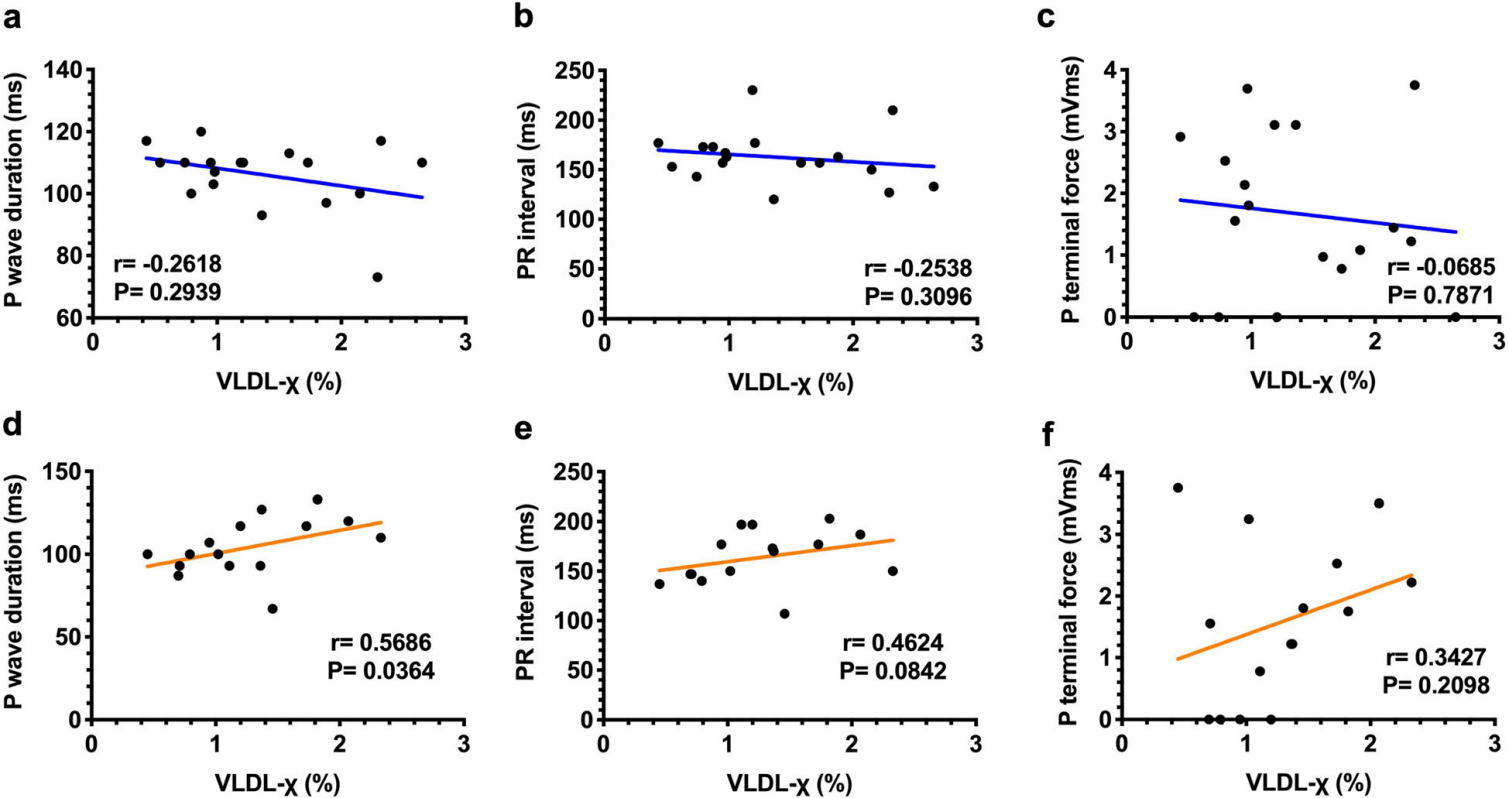
Correlation between electrocardiographic parameters that indicate electrical remodeling of the atria (P wave duration, PR interval and terminal force of P wave) and negatively charged very-low-density lipoprotein as a percentage (VLDL-χ) 2 h after consuming a meal in individuals without (in blue, **a**-**c**, *n* = 15) and with metabolic syndrome (in brown, **d**-**f**, *n* = 15). A significant correlation (with *P* < 0.05) is indicated with each correlation factor and *P* value

Markers of electrical remodeling on P waves were measured and analyzed (Fig. 5a-f). The P wave duration was significantly correlated with 2-h postprandial VLDL-χ in the MetS group (*P* = 0.036; Fig. 5d), and the PR interval tended to be correlated with VLDL-χ (Fig. 5e) but without a significant correlation in the non-MetS group (Fig. 5a-c).

### Different kinetics of VLDL-χ in MetS subjects with larger LA size

**Fig. 6:**
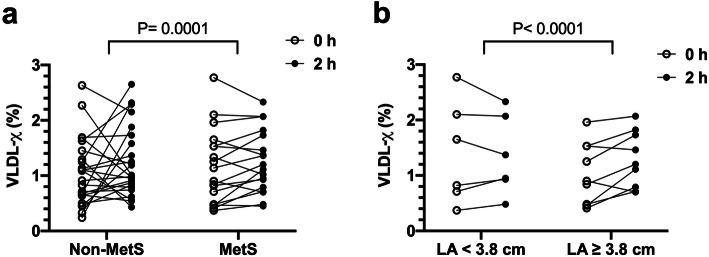
**a** The change in plasma VLDL-χ from fasting (0 h) to 2 h after breakfast in the nonmetabolic syndrome (non-MetS) group was different from that in the MetS group. **b** In the MetS group, subjects with an enlarged left atrial diameter (≥3.8 cm) had exhibited increases in VLDL-χ postprandially

The changes in VLDL-χ from fasting to 2 h after the meal are shown in Fig. 6. The pattern of changes was different between the non-MetS and MetS groups (*P* = 0.0001; Fig. 6a). In the MetS group with dilated LA (diameter ≧ 3.8 cm), VLDL-χ increased after the meal compared with the level in the fasting state, and VLDL-χ remaining steady in those with LA diameters smaller than 3.8 cm (*P* < 0.0001; Fig. 6b).

### VLDL-χ was independently correlated with LA diameter in the MetS group

There was a significant correlation between VLDL-χ and blood pressure and waist and hip circumference. After adjusting for variables of obesity and blood pressure, VLDL-χ showed an independent positive correlation with LA diameter in the MetS group (Table 3). Each 1% increase in VLDL-χ was estimated to increase the LA diameter by 0.23 cm (*P* = 0.028). The other independent determining factor of LA diameter is waist circumference.

**Table 3:** Univariate and multivariable linear regression of determining factors of LA diameter in MetS group

	Univariate linear regression			Multivariable linear regression^a^			
	Regression coefficient	95% confidence interval	*p* value	Regression coefficient	95% confidence interval	T	*p* value
Age	−0.003	(−0.018, 0.012)	0.684				
Hypertension	0.480	(0.298, 0.661)	< 0.001*				
Diabetes mellitus	0.330	(0.137, 0.523)	0.001*				
BMI	0.053	(0.02, 0.087)	0.02*				
Waist circumference	0.027	(0.015, 0.039)	< 0.001*	0.028	(0.015, 0.040)	4.544	< 0.001*
Hip circumference	0.02	(0.004, 0.035)	0.012*				
Systolic BP	−0.002	(−0.008, 0.005)	0.646				
Diastolic BP	0.000	(−0.009, 0.01)	0.949				
Heart rate	−0.005	(− 0.015, 0.005)	0.34				
Triglycerides	< 0.001	(−0.001, 0.002)	0.662				
HDL-C	−0.009	(− 0.024, 0.005)	0.187				
VLDL-χ	0.258	(0.034, 0.482)	0.024*	0.225	(0.025, 0.425)	2.25	0.028*
LVEDD	0.166	(−0.52, 0.385)	0.134				
LVEDV	0.005	(0.002, 0.009)	0.005*				
EF	0.018	(−0.00, 0.037)	0.067				
E/E’ med	−0.015	(−0.067, 0.037)	0.567				
E/E’ lat	−0.008	(−0.085, 0.068)	0.828				
				Model adjusted R-square			
				0.37			

^a^Hierarchical multivariable linear regression in stepwise method with variables including age, hypertension, diabetes mellitus, body mass index (BMI), waist circumference, hip circumference, systolic blood pressure (BP), diastolic BP, heart rate, triglycerides, very-low-density lipoprotein (VLDL)-χ, high-density lipoprotein cholesterol (HDL-C), left ventricle end-diastolic dimension (LVEDD), left ventricle end-diastolic volume (LVEDV), ejection fraction (EF), ratio of mitral flow E velocity and tissue Doppler E’ velocity of the medical and lateral mitral ring, i.e., E/E’ med and E/E’ lat

For all subjects in this study, the univariate analysis revealed four factors that were significantly correlated with LA diameter, including waist circumference, LVEDD, LV end-diastolic volume (LVEDV), and E/E’ med. Among those factors, waist circumference and LVEDD remained independent determinants of LA diameter in multivariable analysis (see Suppl. Table S1). Among non-MetS subjects, age, BMI, and LVEDD were significantly and independently correlated with LA diameter (see Suppl. Tables S2-S3). Similar findings were found for the MetS group after matching our study subjects by age (Suppl. Tables S1 and S3).

## Discussion

The main results from this study are as follows: (1) the postprandial kinetics of glucose, triglycerides, HDL-C, LDL-C, LDL-χ and VLDL-χ were determined; (2) LA dilation was associated with markers of central obesity, hypertension, and postprandial VLDL-χ; (3) 2-h postprandial VLDL-χ was associated with P wave duration in electrocardiography and independently correlated with LA diameter; and (4) MetS subjects whose VLDL-χ increased after the meal had a larger LA diameter than those whose VLDL-χ decreased.

The metabolism of VLDL is an important determinant of LDL formation and triglyceride levels. Both normal and hypertriglyceridemic VLDL molecules contain apoB-100 and apoE. Hypertriglyceridemic VLDL has a higher content of apoE, which facilitates its affinity to VLDL receptors [35]. The tissue distribution of VLDL receptors is quite different from that of LDL receptors. The VLDL receptor is abundant in tissue largely utilizing fatty acids as an energy source, including heart, muscle, and adipose tissue, and it mediates cellular catabolism and regulates the levels of lipoprotein lipase [36, 37]. Whether the modulation of VLDL receptor expression is involved in VLDL-χ-related LA remodeling needs further study.

The importance of postprandial hyperlipidemia in the context of atherosclerosis was suggested early in 1979 [38] and was later increasingly explored in recent years [10, 16, 17]. In 2016, experts reached a consensus regarding the complementary value of nonfasting measurements of lipid profiles for clinical and laboratory medicine [39]. More recently, Nakajima et al. reported evidence to disclose the predictive role of remnant lipoproteins in coronary heart diseases [20]. They also reported that compared to fasting VLDL, postprandial VLDL has a higher affinity for VLDL receptors and easier internalization [40]. Accordingly, it might be suggested that the postprandial VLDL-χ has larger internalization and results in cytotoxicity in atrial tissue and ultimately significant remodeling of the atrium. To the best of our knowledge, our study is the first to report the correlation of postprandial VLDL-χ with LA remodeling.

The association of markers of central obesity with LA size is consistent with results derived by other clinical studies [41, 42]. Obesity, as a well-identified risk factor for AF, causes discrete atrial cardiomyopathy in animal models [43, 44]. Experimental hypertensive rats have fibrosis, inflammation, atrial wavelength shortening and calcium current changes in the LA [45]. Patients with obesity often have coexisting hypertension and dyslipidemia; therefore, it is difficult to identify the major cause of atrial remodeling. In this study, the postprandial VLDL-χ was shown as an independent determinant for LA dilatation.

### Study strength and limitations

This study examined the role of VLDL-χ not only for fasting state but also for postprandial state with blood sampling at different time points related to the unified meal. By the multiple-sampling methods, this study was therefore able to unveil the significant role for VLDL-χ in structural and electrical remodeling of LA, which results in vulnerability to AF. This study also determined the specific time point as of postprandial 2 h, which is coincident with the peak phase for VLDLs on transportation of lipids in circulation after food intake. Here, there are several study limitations to be indicated. First, obtaining postprandial samples in a random manner reduced postprandial data size, and may introduce bias in the results. Second, the VLDL-χ was not determined as of real concentrations. Third, the age between two groups with and without MetS were not matched. This limitation had been overcome by using the age-matching procedure of statistics. Similar multivariable analysis results were found in the matched groups (MetS group: 49.9 ± 7.3 vs. non-MetS group: 49.7 ± 7.2, *P* = 0.89) (in Suppl. Table S1 to S3). Lastly, the impact of age on postprandial VLDL-χ could not be analyzed with relatively small sample size in this study.

## Conclusions

A significant correlation between postprandial plasma VLDL-χ and LA remodeling, particularly in the MetS group, was derived by this study. Despite other factors, such as hypertension and obesity, which also cause LA remodeling in MetS patients, plasma VLDL-χ is still attributed to LA remodeling. We suggest that the metabolism of VLDL and the characteristics of postprandial VLDL-χ are the major determinants of the progression of LA remodeling, particularly in MetS patients. The pathogenesis of AF is complex and can involve a combination of etiological factors and metabolism-associated elements. In many patients, AF is not curable at the time of diagnosis due to long-lasting and overt atrial remodeling with extensive fibrosis. For MetS patients, the association of postprandial plasma VLDL-χ on LA remodeling may give some insights for early prevention of AF.

## Supplementary information

**Additional file 1:** **Table S1.** Univariate and multivariable linear regression of determining factors of LA diameter in the matched groups (*n* = 94). **Table S2.** Multivariable linear regression of determining factors of LA diameter by metabolic syndrome (MetS). **Table S3.** Multivariable linear regression of determining factors of LA diameter by metabolic syndrome (MetS) in the matched groups (*n* = 94). **Figure S1.** Correlation between left atrial (LA) diameter and plasma concentration of glucose before (fasting) and after the unified meal (postprandial 0.5, 1, 2, and 4 h) in participants without MetS (in blue, A-E) and with MetS (in orange, F-J). **Figure S2.** Correlation between left atrial (LA) diameter and plasma concentration of triglyceride before (fasting) and after the unified meal (postprandial 0.5, 1, 2, and 4 h) in participants without MetS (in blue, A-E) and with MetS (in orange, F-J). **Figure S3.** Correlation between left atrial (LA) diameter and plasma concentration of very-low-density lipoprotein cholesterol (VLDL-C) before (fasting) and after the unified meal (postprandial 0.5, 1, 2, and 4 h) in participants without MetS (in blue, A-E) and with MetS (in orange, F-J). **Figure S4.** Correlation between left atrial (LA) diameter and plasma concentration of negative-charged low-density lipoprotein cholesterol (LDL-χ) before (fasting) and after the unified meal (postprandial 0.5, 1, 2, and 4 h) in participants without MetS (in blue, A-E) and with MetS (in orange, F-J).

## Data Availability

All the re-identified data are available upon reasonable request (hclee@kmu.edu.tw).

## Abbreviations

AF: Atrial fibrillation
LA: Left atrium
LV: Left ventricle
LVEF: Left ventricle ejection fraction
MetS: Metabolic syndrome
VLDL: Very low density lipoprotein
VLDL-χ: Negatively charged very low density lipoprotein
