# Supplementary material for: The role of postprandial very-low-density lipoprotein in the development of atrial remodeling in metabolic syndrome
Source: Lipids Health Dis. 2020 Sep 22;19:210. doi: 10.1186/s12944-020-01386-5 (PMC7507670; doi:10.1186/s12944-020-01386-5)
Supplement: Supplementary file 1 — Additional file 1: Table S1. Univariate and multivariable linear regression of determining factors of LA diameter in the matched groups (n = 94). Table S2. Multivariable linear regression of determining factors of LA diameter by metabolic syndrome (MetS). Table S3. Multivariable linear regression of determining factors of LA diameter by metabolic syndrome (MetS) in the matched groups (n = 94). Figure S1. Correlation between left atrial (LA) diameter and plasma concentration of glucose before (fasting) and after the unified meal (postprandial 0.5, 1, 2, and 4 h) in participants without MetS (in blue, A-E) and with MetS (in orange, F-J). Figure S2. Correlation between left atrial (LA) diameter and plasma concentration of triglyceride before (fasting) and after the unified meal (postprandial 0.5, 1, 2, and 4 h) in participants without MetS (in blue, A-E) and with MetS (in orange, F-J). Figure S3. Correlation between left atrial (LA) diameter and plasma concentration of very-low-density lipoprotein cholesterol (VLDL-C) before (fasting) and after the unified meal (postprandial 0.5, 1, 2, and 4 h) in participants without MetS (in blue, A-E) and with MetS (in orange, F-J). Figure S4. Correlation between left atrial (LA) diameter and plasma concentration of negative-charged low-density lipoprotein cholesterol (LDL-χ) before (fasting) and after the unified meal (postprandial 0.5, 1, 2, and 4 h) in participants without MetS (in blue, A-E) and with MetS (in orange, F-J). [file 12944_2020_1386_MOESM1_ESM.docx]

Table S1. Univariate and multivariable linear regression of determining factors of LA diameter in the matched groups (n=94)

|  | Univariate linear regression | | | Multivariable linear regression^#^ | | | |
| --- | --- | --- | --- | --- | --- | --- | --- |
|  | Regression coefficient | 95% confidence interval | p value | Regression coefficient | 95% confidence interval | T | p value |
| Age | 0.006 | -0.011, 0.023 | 0.52 | 0.009 | -0.003, 0.022 | 1.47 | 0.14 |
| Hypertension | 0.52 | 0.29, 0.75 | <0.001* |  |  |  |  |
| Diabetes mellitus | 0.38 | 0.14, 0.62 | 0.002* |  |  |  |  |
| BMI | 0.08 | 0.06, 0.10 | <0.001* |  |  |  |  |
| Waist circumference | 0.029 | 0.02, 0.036 | <0.001* | 0.031 | 0.022, 0.040 | 6.82 | <0.001* |
| Hip circumference | 0.034 | 0.021, 0.047 | <0.001* |  |  |  |  |
| Systolic BP | 0.010 | 0.005, 0.015 | <0.001* |  |  |  |  |
| Diastolic BP | 0.015 | 0.007, 0.023 | <0.001* |  |  |  |  |
| Heart rate | 0.002 | -0.007, 0.012 | 0.61 | -0.005 | -0.012, 0.002 | 1.47 | 0.14 |
| Triglycerides | 0.33 | 0.095, 0.57 | 0.007 | -0.19 | -0.41, 0.02 | 1.75 | 0.08 |
| HDL-C | -0.01 | -0.02, -0.006 | <0.001* |  |  |  |  |
| VLDL-χ | 0.060 | -0.15, 0.27 | 0.57 | 0.19 | 0.04, 0.33 | 2.48 | 0.02* |
| LVEDD | 0.56 | 0.36, 0.77 | <0.001* | 0.33 | 0.14, 0.51 | 3.48 | 0.001 |
| LVEDV | 0.007 | 0.004, 0.010 | <0.001 |  |  |  |  |
| EF | 0.005 | -0.007, 0.016 | 0.45 |  |  |  |  |
| E/E’ med | 0.035 | -0.008, 0.079 | 0.11 |  |  |  |  |
| E/E’ lat | 0.015 | -0.044, 0.074 | 0.61 |  |  |  |  |
|  |  |  |  | Model adjusted R-square | | | |
|  |  |  |  | 0.54 | | | |
| ^#^Hierarchical multivariate linear regression in stepwise method with variables including age, hypertension, diabetes mellitus, body mass index (BMI), waist circumference, hip circumference, systolic blood pressure (BP), diastolic BP, heart rate, triglycerides, very-low-density lipoprotein (VLDL)-χ, high-density lipoprotein cholesterol (HDL-C), left ventricle end-diastolic dimension (LVEDD), left ventricle end-diastolic volume (LVEDV), ejection fraction (EF), ratio of mitral flow E velocity and tissue Doppler E’ velocity of the medical and lateral mitral ring, i.e., E/E’ med and E/E’ lat. | | | | | | | |

Table S2 Multivariable linear regression of determining factors of LA diameter by metabolic syndrome (MetS)

|  | Non-MetS | | | MetS | | |
| --- | --- | --- | --- | --- | --- | --- |
|  | Regression coefficient | 95% confidence interval | p value | Regression coefficient | 95% confidence interval | p value |
| Age |  |  |  |  |  |  |
| Hypertension |  |  |  |  |  |  |
| Diabetes mellitus |  |  |  |  |  |  |
| BMI |  |  |  |  |  |  |
| Waist circumference |  |  |  | 0.026 | 0.016, 0.036 | <0.001* |
| Hip circumference |  |  |  |  |  |  |
| Systolic BP |  |  |  |  |  |  |
| Diastolic BP |  |  |  |  |  |  |
| Heart rate |  |  |  |  |  |  |
| Triglycerides |  |  |  |  |  |  |
| HDL-C |  |  |  |  |  |  |
| VLDL-χ |  |  |  | 0.26 | 0.06, 0.46 | 0.01* |
| LVEDD | 0.73 | 0.52, 0.93 | <0.001* |  |  |  |
| LVEDV |  |  |  |  |  |  |
| EF |  |  |  | 0.015 | <0.001, 0.015 | 0.05 |
| E/E’ med | 0.037 | -0.008, 0.08 | 0.11 |  |  |  |
| E/E’ lat |  |  |  |  |  |  |
|  | Model adjusted R-square | | | | |  |
|  | 0.45 | |  | 0.41 | |  |
| #Hierarchical multivariate linear regression in stepwise method with variables including age, hypertension, diabetes mellitus, body mass index (BMI), waist circumference, hip circumference, systolic blood pressure (BP), diastolic BP, heart rate, triglycerides, very-low-density lipoprotein (VLDL)-χ, high-density lipoprotein cholesterol (HDL-C), left ventricle end-diastolic dimension (LVEDD), left ventricle end-diastolic volume (LVEDV), ejection fraction (EF), ratio of mitral flow E velocity and tissue Doppler E’ velocity of the medical and lateral mitral ring, i.e., E/E’ med and E/E’ lat. | | | | | | |

Table S3 Multivariable linear regression of determining factors of LA diameter by metabolic syndrome (MetS) in the matched groups (n=94)

|  | Non-MetS | | | MetS | | |
| --- | --- | --- | --- | --- | --- | --- |
|  | Regression coefficient | 95% confidence interval | p value | Regression coefficient | 95% confidence interval | p value |
| Age | 0.017 | 0.001, 0.032 | 0.04* |  |  |  |
| Hypertension |  |  |  | 0.34 | 0.008, 0.67 | 0.05 |
| Diabetes mellitus |  |  |  |  |  |  |
| BMI | 0.10 | 0.045, 0.16 | 0.001* |  |  |  |
| Waist circumference |  |  |  | 0.026 | 0.013, 0.039 | <0.001* |
| Hip circumference |  |  |  |  |  |  |
| Systolic BP |  |  |  |  |  |  |
| Diastolic BP |  |  |  |  |  |  |
| Heart rate |  |  |  |  |  |  |
| Triglycerides |  |  |  |  |  |  |
| HDL-C | 0.007 | -0.001, 0.014 | 0.09 |  |  |  |
| VLDL-χ |  |  |  | 0.41 | 0.17, 0.65 | 0.002* |
| LVEDD | 0.42 | 0.11, 0.72 | 0.01* |  |  |  |
| LVEDV |  |  |  |  |  |  |
| EF |  |  |  |  |  |  |
| E/E’ med |  |  |  |  |  |  |
| E/E’ lat |  |  |  |  |  |  |
|  | Model adjusted R-square | | | | |  |
|  | 0.63 | |  | 0.44 | |  |
| #Hierarchical multivariate linear regression in stepwise method with variables including age, hypertension, diabetes mellitus, body mass index (BMI), waist circumference, hip circumference, systolic blood pressure (BP), diastolic BP, heart rate, triglycerides, very-low-density lipoprotein (VLDL)-χ, high-density lipoprotein cholesterol (HDL-C), left ventricle end-diastolic dimension (LVEDD), left ventricle end-diastolic volume (LVEDV), ejection fraction (EF), ratio of mitral flow E velocity and tissue Doppler E’ velocity of the medical and lateral mitral ring, i.e., E/E’ med and E/E’ lat. | | | | | | |

Suppl. Figure 1

**Suppl. Figure 1.** Correlation between left atrial (LA) diameter and plasma concentration of glucose before (fasting) and after the unified meal (postprandial 0.5, 1, 2, and 4 hours) in participants without MetS (in blue, A-E) and with MetS (in orange, F-J).

Suppl. Figure 2

**Suppl. Figure 2.** Correlation between left atrial (LA) diameter and plasma concentration of triglyceride before (fasting) and after the unified meal (postprandial 0.5, 1, 2, and 4 hours) in participants without MetS (in blue, A-E) and with MetS (in orange, F-J).

Suppl. Figure 3

**Suppl. Figure 3.** Correlation between left atrial (LA) diameter and plasma concentration of very-low-density lipoprotein cholesterol (VLDL-C) before (fasting) and after the unified meal (postprandial 0.5, 1, 2, and 4 hours) in participants without MetS (in blue, A-E) and with MetS (in orange, F-J).

Suppl. Figure 4

**Suppl. Figure 4.** Correlation between left atrial (LA) diameter and plasma concentration of negative-charged low-density lipoprotein cholesterol (LDL-χ) before (fasting) and after the unified meal (postprandial 0.5, 1, 2, and 4 hours) in participants without MetS (in blue, A-E) and with MetS (in orange, F-J).
